# High frequency regeneration of plants via callus-mediated organogenesis from cotyledon and hypocotyl cultures in a multipurpose tropical tree (*Neolamarkia Cadamba)*

**DOI:** 10.1038/s41598-020-61612-z

**Published:** 2020-03-12

**Authors:** Hao Huang, Ying Wei, Yongjin Zhai, Kunxi Ouyang, Xiaoyang Chen, Longhua Bai

**Affiliations:** 1 Guangxi Botanical Garden of Medicinal Plants, Nanning, 530023 China; 2 College of Forestry and Landscape Architecture, South China Agricultural University, Guangzhou, 510642 China

**Keywords:** Agricultural genetics, Molecular engineering in plants

## Abstract

In this works, a simple, efficient and repeatable protocol was developed for *in vitro* regeneration via callus-mediated organogenesis of *Neolamarkia Cadamba* using cotyledonary petioles and hypocotyls. Effects of basal medium, plant growth regulators, the types and age of explant on the formation of adventitious buds/shoots were studied. Meanwhile, histological analysis for early ontogenic stages and genetic stability assessment by flow cytometry were investigated. Our investigation demonstrated that, compared with 6-benzyladenine (BA), N^6^-(2-isopentenyl) adenine (2-ip), Thidiazuron (TDZ) was the optimal cytokinin for buds/shoots induction on cotyledon and hypocotyl explants. Douglas-fir and sugar pine medium (DCR) supplemented with 22.7 μM TDZ and 0.27 μM α-naphthalene acetic acid (NAA) was most effective on bud induction, with the highest bud-induction rate and numbers of buds on cotyledon and hypocotyl explants. The available shoot per explant hit 35.2 when the induced callus sub-cultured to a medium without TDZ. It was found that TDZ could promote induction of the callus and the buds, however, continuous exposure beyond 4 weeks of supplemented high concentration (exceed 11.35 μM), TDZ was harmful to the proliferation and growth of buds/shoots. DCR appeared more efficiency than Murashige and Skoog medium (MS), Woody Plant medium (WPM), anther culture of cereal crops medium (N_6_) on bud induction. Age of cotyledon and hypocotyl explants in 20-day to 25-day was most beneficial to adventitious buds/shoots formation. Histological investigation confirmed that the buds originated from the wounded incisions of cotyledonary petiole and hypocotyl fragments, with callus formation. The regeneration plantlets were successfully acclimatized in greenhouse, yielded above 95% survival rate in field, exhibited normal morphology and growth characteristics. The analysis of flow cytometry on *N. cadamba* indicated no variation in the ploidy levels between the regenerated plantlets and the donor trees. The developed procedure can be used for mass production, germplasm exchange and transgenic studies to improve the resistance of the species via *Agrobacterium*-mediated.

## Introduction

*Neolamarckia cadamba* (commonly known as burflower tree, kadamba tree or kadamb) is a tropical tree of great economic importance. First, *N. cadamba* trees grow fast, and their wood is good for pulp production, furniture, and other construction purposes^1^. Second, *N. cadamba* has been traditionally used as medicine to treat diseases such as dysentery, fever and snake bites ^2,3^. Now, from its bark, leaves, and flowers people extract phytochemical compounds, including monoterpenoid, triterpenoid saponin, and ethylene glycol^4–9^, alkaloid^10^, cadambine^7,11^, and use them to treat hyperglycemia^12^, hyperlipidemia^13,14^, hypertension^15^, and wound and skin diseases^16^. Third, *N. cadamba* is a desirable landscape tree, and is suitable for reforestation programs. Because it grows extremely fast and all parts of the tree can bring profits to the growers, *N. cadamba* is called “miracle tree” and “gems tree”.

*N. cadamba*, however, is susceptible to many biotic and abiotic stresses, and needs improvement. For example, because they are juicy and nutritious, buds and young leaves are often attacked by Lepidoptera and Coleoptera insects such as *Dianhania glauculelis* and *Acalolepta cervina*^17^. Because they are especially sensitive to frost, currently *N. cadamba* trees are broadly planted only in India, Nepal, Thailand, Malaysia, Papua New Guinea and warm areas of China^18,19^. In order to make it more profitable to grow *N. cadamba* trees, breeding to improve important agronomic traits becomes increasingly important.

Since *N. cadamba* trees produce seeds through cross-pollination and seeds take above 5 years to grow into mature trees that start to flower, breeding *N. cadamba* using conventional methods will be too slow. Therefore, to shorten its breeding process, we must develop and adopt new techniques. One such technique is micropropagation, which can rapidly produce clones of selected individuals that carry the desired traits and thus save years on segregation and selection. Protocols for micropropagating *N. cadamba* is now available^20–24^, although further refinement is needed for the protocols to be effective on broader genotypes and explant sources. Another way to speed up *N. cadamba* breeding is genetic engineering. By using genetic engineering techniques, we can deliver trait-determining genes into somatic cells and from transformed cells regenerate trees with the desired traits, a procedure that may be completed in months instead of the many years that a single sexual reproduction cycle takes. Genetic engineering includes multiple steps. For its successful use in the improvement of *N. cadamba*, a key step is genetic transformation, which requires an efficient regeneration protocol^25^.

Previously, we reported a procedure for adventitious buds/shoots induction from the cotyledons of *N. cadamba*^21^. The directly regeneration protocol is ideal for micropropagation to obtain a large number of elite seedlings, because the buds/shoots originate directly from epidermal cells, bypassing a prominent callus stage and thus reducing the chance of epigenetic variation^26^. The protocol, however, is not suitable for genetic transformation (based on this directly regeneration protocol, we attempted to do many genetic transformation experiments, but all failed), which usually includes a step of selection for transformations at the callus-induction stage. Therefore, we set about developing a new protocol that would be easily adapted for genetic transformation. We first tested and identified plant growth regulators that could efficiently induce adventitious buds/shoots through callus-mediated organogenesis, and then optimized the protocol by testing different basal media, subculture media and types of explants. Last, we tested procedures for root induction, acclimation, ploidy of regenerated plants.

## Materials and Methods

### Explant sources

We collected mature seeds from a *N. cadamba* tree of over 10 years old in Guangxi Botanical Garden of Medicinal Plants (Nanning, China). To promote germination, we first soaked seeds in 40 °C water for 24 h, by placing the container on a thermostat shaker set to 40 °C and 120 rpm. Then we sterilized the seeds with 20% bleach (5.0% sodium hypochlorite) for 15 min, rinsed them with water for three times, and planted them on MS agar medium supplemented with 3.0% (w/v) sucrose and 0.6% (w/v) plant agar. Seeds germinated, and seedlings grew under this condition: 25 ± 2 °C; 14-h photoperiod of 90 μmol m^−2^ s^−1^ irradiance; and relative humidity of 70%. From 20-day to 45-day seedlings we collected cotyledons (with or without cotyledonary petiole) and hypocotyl fragments (3–4 mm in length), and used them as explants.

### Adventitious buds/shoots induction

To induce adventitious buds/shoots, we cultured cotyledon explants (abaxial side touching medium surface) and hypocotyl fragments (flat on medium surface) on different induction media under this condition: 25 ± 2 °C; 14-h photoperiod of 90 μmol m^−2^ s^−1^ irradiance; and relative humidity of 70%. All induction media were supplemented with 3.0% (w/v) sucrose and 0.6% (w/v) plant agar, and their pH was adjusted to 5.8. Media were autoclaved at 121 °C for 20 min, cooled to ~60 °C before adding plant growth regulators (PGRs), and aliquoted into sterile 250-ml flasks (~25 ml each). The PGRs used in this study (2-ip, TDZ, BA and NAA) were purchased from Beijing Dingguo Changsheng Biotechnology Co., Ltd (Beijing, China). The basal media (MS, DCR, N_6_, WPM) were prepared according to original publications^27–30^.

### Root induction and plantlet acclimatization

Procedures follow Huang *et al*.^21^. Briefly, elongated adventitious shoots (3–4 cm) were transferred to MS agar medium supplemented with 0.25 μM IBA and 0.27 μM NAA, and cultured at 25 ± 2 °C, first in dark for 2 days and then under a 12-h photoperiod (90 μmol m^−2^ s^−1^) for 8 days. For acclimatization, flasks containing plantlets were moved from culture room to greenhouse and kept there for 3–4 days. The plantlets were then removed from flask and transferred to soil.

### Paraffin sections

To trace the early ontogenic stages of bud regeneration, samples were periodically taken and fixed in FAA solution^30^ (37% formalin: glacial acetic acid: 50% ethanol, ratio 5:5:90, in volume) until the adventitious buds were discernible with the naked eye. The samples were progressively dehydrated in a graded ethanol series (70–100%), then embedded in paraplast and mounted on block-holders. Paraffin blocks were sectioned into 8-μm slices with a Reichert 820 H Histostat rotary microtome (Warer-Lambert Tech. Inc., USA). The sections were affixed to slides, stained with fast green, covered with a cover slip in place with help of a thin coating of Neutral Balsam, and then dried at 38 °C for 48 h^30^. All sections were observed and photographed with a Leica DMLB microscope (Leica, Inc. Germany).

### Assessment of the ploidy of regenerated plants

Young leaves from regenerated plants and donor plants were collected, and their DNA contents were analyzed using a flow cytometer (BD Accuri C6 Plus, USA). Sample process and operation followed the instruction manual. Software CFlow Plus was used to analyze the data and generate the figures.

### Statistical analysis

Data from all experiments were subjected to ANOVA. The means were compared using software SPSS (version 19.0) to carry out Duncan’s multiple range test.

## Result and Discussion

### Effects of PGRs on induction of adventitious buds/shoots

In our previous study^21^, the most efficient bud/shoot induction was achieved by culturing cotyledon explants on DCR medium supplemented with synthetic plant hormones NAA (0.27 μM) and BA (22.22 μM). In this study, we continued to use DCR medium supplemented with a fixed concentration (0.27 μM) of auxin NAA, but tested different concentrations of 3 cytokinins: BA, 2-ip and TDZ. In addition to cotyledons, we also tested hypocotyl fragments as explants. At the end of 4-week culture, we counted and calculated the percentages of explants that produced adventitious buds (originated from callus, about 0.3 cm in length) and the average number of shoots (originated from the adventitious buds above 0.7 cm length) per explant.

Plant hormone cytokinins are divided into two main types: adenine-type cytokinins and phenylurea-type cytokinins. Both BA and 2-ip belong to the first group, and they showed similar effects in our study. First, at concentration of 22.22 μM, BA induced adventitious buds on 52.4% of cotyledon explants, and each explant produced, on average, 4 shoots (Table 1), a result that was comparable with our previous observations, 54.0% and 4.4 shoots^21^. Second, also at its optimum concentration (12.30 μM), 2-ip induced adventitious buds on 49.8% of cotyledon explants, although explants produced fewer shoots, only 1 shoot per explant (Table 1). Third, similar to BA, 2-ip induced buds/shoots to develop directly from the epidermal cells at the edge of the cuts, without an intermediate stage of callus (data not shown). Based on these observations, we concluded that BA and 2-ip did not suit our purpose.

**Table 1:** Effect of PGRs on bud induction from explants of *N. cadamba* observed after 4weeks.

PGRs(μM)				Cotyledons		Hypocotyls	
2-ip	TDZ	BA	NAA	Bud induction (%)	Number of shoots/explants	Bud induction (%)	Number of shoots/explant
0.00	0.00	0.00	0.00	0.0 ± 0.0^F^	0.0 ± 0.0^E^	0.0 ± 0.0^F^	0.00^E^
0.00	0.00	0.00	0.27	0.0 ± 0.0^F^	0.0 ± 0.0^E^	0.0 ± 0.0^F^	0.00^E^
2.46	0.00	0.00	0.27	0.0 ± 0.0^F^	0.0 ± 0.0^E^	0.0 ± 0.0^F^	0.0 ± 0.0^E^
4.92	0.00	0.00	0.27	25.6 ± 1.9^E^	0.0 ± 0.0^E^	0.0 ± 0.0^F^	0.0 ± 0.0^E^
12.30	0.00	0.00	0.27	49.8 ± 1.6^C^	1.0 ± 0.0^D^	9.4 ± 0.4^E^	0.5 ± 0.3^D^
24.61	0.00	0.00	0.27	32.6 ± 1.4^D^	0.9 ± 0.2^D^	7.2 ± 4.8^D^	0.3 ± 0.1^C^
0.00	2.27	0.00	0.27	60.5 ± 2.4^B^	1.4 ± 0.2^CD^	72.9 ± 2.4^C^	1.3 ± 0.1^C^
0.00	4.54	0.00	0.27	100.0 ± 0.0^A^	1.9 ± 0.1^C^	88.6 ± 1.8^B^	1.3 ± 0.2^C^
0.00	11.35	0.00	0.27	100.0 ± 0.0^A^	2.5 ± 0.1^BC^	100.0 ± 0^A^	2.8 ± 0.1^B^
0.00	22.70	0.00	0.27	100.0 ± 0.0^A^	2.9 ± 0.2^B^	100.0 ± 0^A^	3.3 ± 0.8^A^
0.00	0.00	2.22	0.27	0.0 ± 0.00^F^	0.0 ± 0.0^E^	0.0 ± 0.0^F^	0.0 ± 0.0^E^
0.00	0.00	4.44	0.27	21.9 ± 0.5^E^	1.8 ± 0.3^C^	0.0 ± 0.0^F^	0.0 ± 0.0^E^
0.00	0.00	11.11	0.27	23.8 ± 0.3^E^	2.4 ± 0.4^BC^	0.0 ± 0.0^F^	0.0 ± 0.0^E^
0.00	0.00	22.22	0.27	52.4 ± 2.5^C^	4.0 ± 0.2^A^	0.0 ± 0.0 ^F^	0.0 ± 0.0^E^

Note: Values represent mean ± SE, n = 4. Data having the same letter in a column were not significantly differed by Duncan’s multiple comparison test (P < 0.01).

Different from BA and 2-ip, TDZ is a phenylurea-type and potent cytokinin for plant tissue culture^31^. The biological action of TDZ has been suggested to be superior or similar to that of the most active adenine-type cytokinins^32^. It plays a role in modulation of the endogenous PRGs either directly or as a result of induced stress^33,34^, and has been shown to be the most critical factor for somatic embryogenesis induction and buds/shoots regeneration^35,36^.

In *in vitro* regeneration of *N. cadamba*, we found that TDZ was far more effective in inducing adventitious buds of *N. cadamba*, and deduced that the bud formation was through callus-mediated organogenesis. On cotyledon explants and for all tested concentrations except the lowest one (2.27 μM), the percentage of bud-induction was 100% (Table 1). On hypocotyl explants and for the two higher concentrations (11.35, 22.70 μM), the percentage of induction also reached 100% (Table 1). At all concentrations tested, TDZ induced shoot formation on both cotyledon and hypocotyl, and the average numbers of shoots per explant ranged from 1.3 to 3.3, with no obvious difference between the two types of explants.

This effect of TDZ is in stark contrast to that of BA and 2-ip, which could effectively induce the formation of buds/shoots on cotyledon explants, but had no or little effect on hypocotyl explants (Table 1). This contrast hinted that TDZ-induced buds did not arise directly from the cells of cotyledon or hypocotyl, two plant organs that differ obviously in shape and conceivably in cell states and the competency for bud formation, instead, they had to arise from calluses–cell masses that originate from different explants but have similar cell states and thus similar rates of buds/shoots formation.

**Figure 1:**
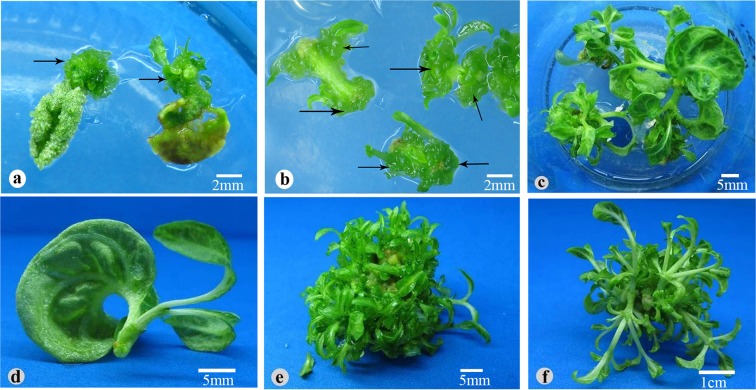
Effect of adventitious bud formation from *N. cadamba* on DCR medium containing 11.35 μM TDZ and 0.27 μM NAA. Callus (arrow) developed from the cotyledons (**a**) and hypocotyls (**b**) after 4 weeks of incubation. (**c**,**d**) Adventitious buds exhibited abnormal and the leaf became involute after 6 weeks of culture. After 4 weeks of culture, subculture on DCR medium containing 22.70 μM BA and 0.27 μM NAA in 20 d (**e**), 30 d (**f**).

Indeed, we observed that clusters of calluses started to emerge from the incisions of cotyledonary petioles and hypocotyl fragments at day 6, and, after continued culture on the same TDZ-containing medium, on some calluses developed adventitious buds (Fig. 1c,d). We sampled explants at different culture stages and did histological analysis. We confirmed that the calluses were truly unstructured cell masses and adventitious buds arose randomly from calluses, clearly different from the bud formation induced by BA or 2-ip, which was directly from epidermal cells^21^. Therefore, cytokinin TDZ well suited our purpose, and next we needed to choose a concentration.

Among the 4 TDZ concentrations, as shown in Table 1, 22.7 μM is most effective in inducing adventitious buds/shoots. However, on medium containing 22.7 μM TDZ, many adventitious buds refused to grow after formation and elongated shoots grew involute leaves (Fig. 1c,d). The problem became more severe after extended culture (>6 weeks). On medium containing 11.35 μM TDZ, the problem was less prominent, but the average number of shoots per explant was lower. Liu *et al*.^37^ also reported the similar observations on *Jatropha curcas* that both the concentration and the exposure duration of TDZ on explants influence shoot proliferation and elongation. And, in the *in vitro* regeneration of *Rauvolfa tetraphylla*^38^, *Vitex trifolia*^39^, it was found that prolonged exposure of the culture to TDZ had an adverse effect, too.

To solve the problem and to optimize the protocol, we used two strategies: (1) culture for 4 weeks on medium containing 22.7 μM TDZ to induce adventitious buds, and then subculture on medium containing a lower concentration of TDZ or a different cytokinin for buds to elongate; meanwhile, (2) fix TDZ concentration to 11.35 μM and test other factors that may affect efficiency: basal medium and explant type and age.

### Effect of cotyledonary petiole and explant age on the formation of adventitious buds

When cotyledon explants were cultured on TDZ-containing medium, calluses consistently emerged from the cut zone of the petiole and, less frequently, they also formed on the lower epidermis (also the side touching medium surface) of cotyledon. On the epidermis-derived calluses, however, we never saw adventitious buds. This phenomenon prompted us to compare cotyledon explants with and without petioles. As shown in Table 2, without petioles, calluses could still form on 100% of the cotyledon explants, but none developed adventitious buds. This result and our previous studies^21^ suggested that it was key to include with the cotyledon explant a section of the petiole. The cotyledon explants without petioles could not be induced to buds/shoots formation in the induction medium with only plant hormone cytokinins and NAA.

**Table 2:** Effects of bud formation in different types of cotyledon explants of *N. cadamba*.

Types of cotyledon explants	Callus formation	Bud formation (%)	Number of bud/explants
including petiole	100.0 ± 0.0^A^	100.0 ± 0.0^A^	2.7 ± 0.3^A^
without petiole	100.0 ± 0.0^A^	0.0 ± 0.0^B^	0.0 ± 0.0^B^

Note: value represents the mean ± SE of 4 independent experiments with 30 explants per treatment. Data having the same letter in a column were not significantly differed by Duncan’s multiple comparison test (P < 0.01). DCR medium supplemented with 11.35 μM TDZ and 0.27 μM NAA. Data were collected after 4 weeks of culture.

**Figure 2:**
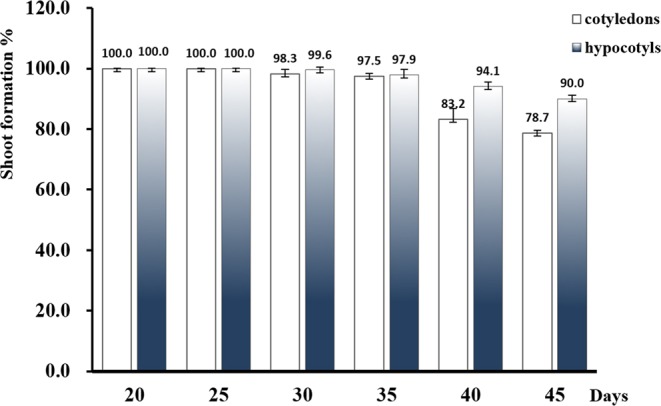
Effects of buds/shoots formation in different age of cotyledon and hypocotyl explants.

As shown above, calluses derived from cotyledons without petioles did not give rise to adventitious buds, suggesting a strong influence of the type of explants. We wondered whether bud formation was also affected by the age of explants. Therefore, we collected cotyledon and hypocotyl explants from seedlings of different ages, and determined their shoot formation rates on DCR medium containing 11.35 μM TDZ and 0.27 μM NAA. Surprisingly, the rate was not significantly affected by the age in 21-day to 35-day; the induction rate remained above 97.0% when explants reached 35-day old, and the lowest percentage (78.7%) occurred on cotyledon in 45 days (Fig. 2). Moreover, result hinted that buds/shoots formation appeared gradually decrease when the explants got older (after 25-day), perhaps cell dedifferentiation became more difficult on the induction medium. In our research, we continued to use explants from 20-day to 25-day seedlings, because they were not only easier to collect than the smaller explants of about 2 weeks old, but also could produce more buds/shoots than the explants of above 30-day old (data not shown).

### Effect of basal medium on the formation of adventitious buds/shoots

When determining that cytokinin TDZ, in combination with auxin NAA, could induce adventitious buds on 100% of explants, we did all tests on DCR medium. We wondered whether the number of buds per explant could be increased by using different media. For this purpose, we fixed the concentrations of TDZ and NAA to 11.35 μM and 0.27 μM, and compared the effects of 4 commonly used basal media, DCR, MS, N_6_ and WPM. As shown in Table 3, basal medium indeed had a profound effect on bud regeneration. Medium DCR was most effective, with bud induction rate of 100% and the highest numbers of buds on both cotyledon and hypocotyl explants. As DCR, MS medium also had bud induction rate of 100%, but produced fewer buds than DCR. Basal media N_6_ and WPM had significantly lower bud induction rates and produced fewer buds. Therefore, basal medium DCR was the best choice, and then followed by **MS**, N6, WPM. It was similar to the publication of Joshi *et al*.^23^, that is, on three basic medium (MS, B5 and White), they found that **MS** medium was more suitable for the bud break and shoots developed, too. These results showed that different basic medium had a significant effect on buds/shoots formation, which was probably due to the difference of chemical element content in different basic medium.

**Table 3:** Effects of basal medium on adventitious bud induction evaluated after 4 weeks.

Basal medium	Cotyledons		Hypocotyls	
	Bud induction (%)	Number of buds/explants	Bud induction (%)	Number of buds/explants
MS	100.0 ± 0.0^A^	2.1 ± 0.3^B^	100.0 ± 0.0^A^	2.4 ± 0.5^B^
DCR	100.0 ± 0.0^A^	2.5 ± 0.1^A^	100.0 ± 0.0^A^	2.8 ± 0.3^A^
N_6_	86.7 ± 0.4^B^	1.7 ± 0.9^C^	89.0 ± 1.6^B^	2.0 ± 0.7^C^
WPM	56.0 ± 1.6^c^	1.2 ± 0.8^D^	64.6 ± 2.1^C^	1.6 ± 1.1^D^

Note: each value represents the mean ± SE of 4 independent experiments with 40 explants per treatment. Data having the same letter in a column were not significantly differed by Duncan’s multiple comparison test (*P* < 0.01). All basal medium supplemented with 11.35 μM and 0.27 μM NAA.

### Effect of subculture medium on TDZ-induced adventitious buds/shoots

We had found that 22.70 μM TDZ, in combination with 0.27 μM of NAA, was most effective in inducing adventitious buds and shoots, but many buds refused to grow after formation and elongated shoots were morphologically abnormal. We solved this problem by using a simple strategy–subculture. To ensure efficient bud induction, explants were first cultured for 4 weeks on 22.70 μM TDZ-containing medium, and then, for the adventitious buds to grow, explants were subcultured for another 3 to 4 weeks on a fresh medium, which contained a lower concentration of TDZ, no hormones, or a different cytokinin. As shown in Table 4, when TDZ concentration was reduced to 9.08 μM, subculture yielded 8.9 shoots per explant, a significant increase from the ~3 shoots produced during initial culture, and the high concentration TDZ-caused bud/shoot abnormalities diminished. When hormones were entirely eliminated, subculture yielded 7.6 shoots per explant, also a significant increase, and furthermore, shoots from the hormone-free subculture medium were perfectly normal. The most striking results, however, were obtained when TDZ was replaced with 22.70 μM BA. On this subculture medium, not only shoots grew normal (Fig. 1e,f)), the average number of available shoots per explant (35.2) also reached unprecedentedly high. The new shoots originated from both the adventitious buds formed during initial culture and the auxiliary buds of adventitious shoots. Similar effect has been reported in several species regeneration including *Phalaenopsis*^40^, *Diospyros kaki*^41^ and *Cicer arietinum*^42^.

**Table 4:** Effect of DCR subculture medium on TDZ-induced adventitious buds/shoot.

PGRs(μM)			Buds induction(%)	Available shoots/ explant	Explants status
TDZ	BA	NAA			
9.08	0.00	0.27	100.0 ± 0.0^A^	8.9 ± 0.7^B^	Callus loosen, granular and adventitious bud points on surface; bud, leaf deformity.
0 0.00	0.00	0.00	100.0 ± 0.0^A^	7.6 ± 0.5^C^	Shoot, leaf grow normal, bud quality generally.
0.00	22.20	0.27	100.0 ± 0.0^A^	35.2 ± 0.6^A^	Callus densification, differentiate into tufted, robust bud; bud, leaf grow normal.

Note: Value represents the mean ± SE of 3 independent experiments with 40 explants per treatment. Data in the same column followed by different letters are significantly different (Duncan’s test at *P* < 1% level).

### Shoot propagation, root formation and acclimatization

**Figure 3:**
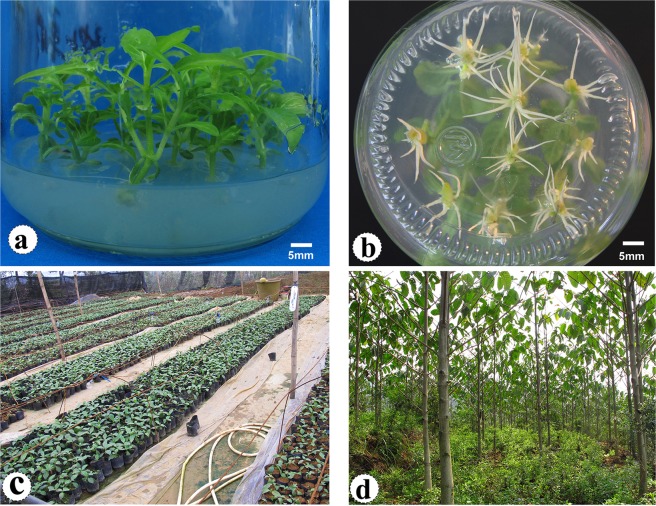
*In vitro* propagation, rooting, acclimatization, and afforestation of *N. cadamba*. (**a**) Shoot propagation. (**b**) Root formation in root initiation medium after 10 days. (**c**) Acclimatization of plantlets in the greenhouse. (**d**) plantlets grew in the field (13-months). Bar: 5 mm.

In these sections, we carried out in accordance with the protocol of our previous publication^21^, the result showed that shoot propagation was achieved successively on the MS medium containing 4.44 μM BA and 0.25 μM IBA, the shoots grew robust, well elongate and more adventitious shoots. The 1/2MS medium containing 0.25 μM IBA and 0.27 μM NAA was the most effect for *in vitro* rooting, root induction rates and numbers of roots were 95.4% and 6.0, respectively. The plantlets yielded above 95% successful acclimatization rate, and grew in the field with normal phenotypes (Fig. 3).

### Histological investigations

**Figure 4:**
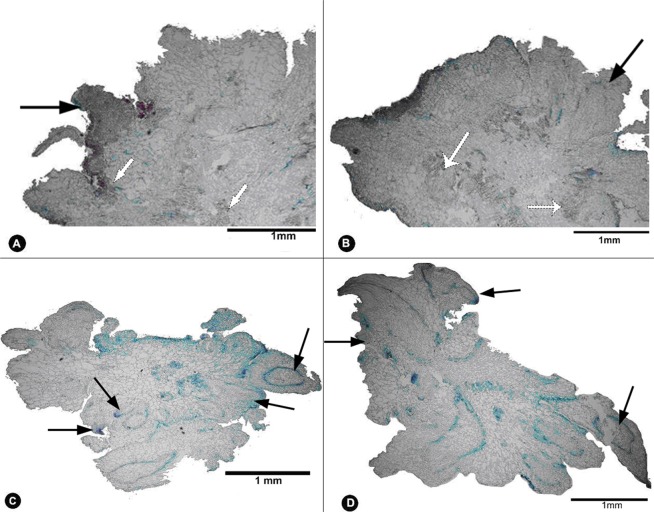
Histological analysis of bud regenerated of *N. cadamba* on DCR medium contained 11.35 μM TDZ and 0.27 μM NAA. Callus (arrow) induced from cotyledonary petioles (**A**) and hypocotyls. (**B**) Adventitious buds (arrow) appeared around cotyledonary petioles (**C**) and hypocotyls (**D**) on 25 days. Bar: 1 mm.

Histological analysis provided morphological details that help explain the process of organogenesis from the explants^43^. At different regeneration stages of explants, it was found that the incision of explants was the source of callus and organs.No significant histological changes were observed during the first 5 days. The first distinct change was that the cells dedifferentiated along the incision of cotyledon and hypocotyl. After 3 weeks of culture, the clusters of callus structures were formed, a few developed adventitious buds/shoots, but most callus were to maintain clusters of callus structures, and the origin of the bud was random happened from the callus (Fig. 4). From the histological analyses on this experiment and our previously study^21^, it was indicated that the regeneration process on DCR medium contained TDZ and NAA was indirect organogenesis pattern.

### The regenerated plants ploidy assessment by flow cytometry

**Figure 5:**
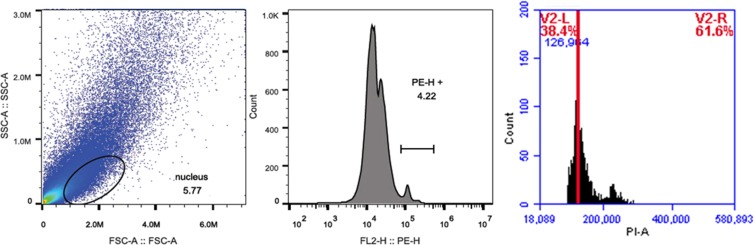
Nuclear DNA contents detection of leave from donor tree.

**Figure 6:**
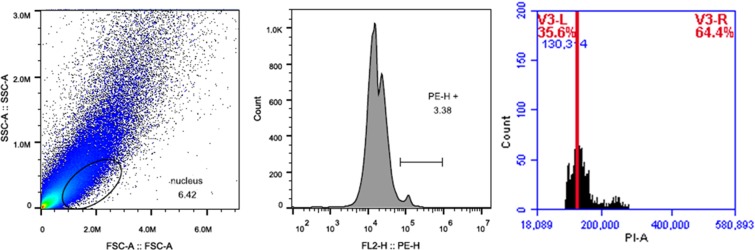
Nuclear DNA contents detection of leave from *in vitro*-derived regenerated plant.

In the regeneration approach of this study, we used sterile cotyledon and hypocotyl as explants to induce adventitious buds/shoots. To verify the effectiveness of this method, Flow cytometry were used to detect the similarities in the DNA ploidy levels between the donor tree and the regenerated plant. The result showed that the peak of fluorescence intensity was approximately equal, the fluorescence intensity of donor tree and induction plantlet were 126964 and 130314, respectively (Figs. 5 and 6). The values indicated, under the condition of *in vitro* culture, there was good genetic stability between the donor tree and the regenerated plant without any somaclonal variation. Furthermore, it was found that these plantlet phenotypic characteristics had no different from the donor plant after they grew on field in 1.5a.

## Conclusion

The study demonstrates, for the first time, an improved, reproducible and highly efficient buds/shoots regeneration protocol of *N. cadamba* was developed in both cotyledonary petiole and hypocotyl explants, used the cytokinin TDZ via an intermediate callus formation. Obviously, in terms of proliferation multiple and potential genetic transformation ability, this *in vitro* regeneration strategy is superior to our previous research^20^. In addition, this is the first report of genetically sustainable proliferation via cotyledonary petiole and hypocotyl explants in *N. cadamba*. it would facilitate successful approach for the mass production of genetically consistent plants, *ex situ* conservation of elite germplasm of this sensitive, multipurpose tree in the future. Furthermore, this simple, efficient and reproducible regeneration strategy of regeneration can be applied to transgenic studies to improve the resistance of the species via *Agrobacterium*-mediated.
